# Welcome to *Tumour Virus Research*

**DOI:** 10.1016/j.tvr.2021.200211

**Published:** 2021-01-27

**Authors:** Peter L. Stern, Lawrence Banks

**Affiliations:** Manchester Cancer Research Centre, University of Manchester, Manchester, M20 4GJ, United Kingdom; Tumour Virology Group, ICGEB, AREA Science Park, Trieste, 34149, Italy

Over the last 50 years the field of *Tumour Virus Research* has provided fundamental insights into the mechanisms underlying tumour development. These studies have, in turn, not only driven the field of tumour virology, but have provided unique insights into many of the fundamentals of cell biology and of the events that ultimately lead to tumourigenesis. This has led to the development of new means of prevention and therapeutic intervention in a wide range of different human malignancies, and not only in those with a viral aetiology.

In the revamping and renaming of *Papillomavirus Research* to *Tumour Virus Research*, we expect the Journal to be the ideal forum for publishing such studies, resulting in an extension of the scope and impact of the work that is published in the Journal. This, in turn will ensure that *Tumour Virus Research* will be more broadly applicable to the wider scientific community working on all human tumour viruses and associated model systems.

To achieve this, we need to ensure the studies we publish are timely, scientifically and technically excellent, and multidisciplinary. To reflect these new goals, *Tumour Virus Research* now has a much broader editorial board, and we welcome submissions from scientists working on all aspects of human tumour viruses.

